# Rapid Fabrication of Bioinspired Compound-Eye Array with Hydrophobicity and Antireflectivity

**DOI:** 10.3390/biomimetics11070507

**Published:** 2026-07-19

**Authors:** Zirui Yao, Lelai Yuan, Jiabao Lu, Gang Huang, Zihao Li, Yu Li, Heng Xie, Guizhen Zhang

**Affiliations:** 1 National Engineering Research Center of Novel Equipment for Polymer Processing, School of Automation Science and Engineering, South China University of Technology, Guangzhou 510640, China; 2 Hubei Key Laboratory of Plasma Chemistry and Advanced Materials, School of Materials Science and Engineering, Wuhan Institute of Technology, Wuhan 430205, China

**Keywords:** compound-eye array, microlens, nanopillar, bioinspired

## Abstract

A strategy combining imprinting with anode oxidation is proposed for preparing an aluminum template with a negative compound-eye array. Injection compression molding with the aluminum template mounted on the mold cavity surface is applied to fabricate polystyrene replicas with a biomimetic compound-eye array on their surfaces. It is demonstrated that orderly microlenses and dense nanopillars with average diameters of approximately 225 μm and 63 nm, respectively, are formed on the polystyrene replicas. The polystyrene replica surfaces with the compound-eye array exhibit both hydrophobicity, with a water contact angle of 151 ± 2° and a rolling angle of 4 ± 1°, and excellent antireflectivity, showing an average reflectance of approximately 4% across the 400–1000 nm wavelength range. The microlens and nanopillar structures on the PS replicas are therefore key to achieving both hydrophobicity and antireflectivity simultaneously. The proposed fast mass-replication approach, which combines imprinting, anode oxidation, and injection compression molding, offers an efficient route for producing bioinspired compound-eye arrays. This strategy shows potential for applications in optoelectronics, photovoltaics, and self-cleaning optical surfaces.

## 1. Introduction

Bioinspired compound-eye structures have garnered tremendous attention due to their exceptional optical properties and broad applicability in photovoltaic, optical, and imaging systems, including solar cells, light-emitting devices, displays, and photodetectors [1,2,3,4]. The unique hierarchical architecture of natural compound eyes, which combines ordered microlens arrays with anti-reflective nanostructures, offers significant advantages in light collection efficiency, wide-angle field of view, and surface wettability [5,6]. These multifunctional characteristics make them highly attractive templates for the design of next-generation functional surfaces in optoelectronics and imaging technologies [7,8]. Consequently, faithfully replicating such bioinspired architectures on polymer substrates holds great promise for improving the optical efficiency, environmental durability, and functional integration of portable electronics, greenhouse photovoltaics, and wearable optical sensors.

To date, a variety of fabrication techniques have been developed to replicate such intricate structures. Nanoimprint lithography has emerged as a high-throughput method capable of sub-100 nm resolution over large areas [9,10]. Soft lithography offers flexibility in patterning complex three-dimensional geometries using elastomeric stamps [11,12]. Electron-beam lithography provides ultrahigh resolution down to the nanometer scale but is inherently slow and costly, making it unsuitable for large-scale production [13,14]. Self-assembly approaches enable low-cost fabrication of ordered micro/nanostructures, yet they often suffer from limited control over structural precision and long-range order [15,16]. These methods can be broadly classified into bottom-up and top-down approaches [17,18]. Bottom-up methods, such as self-assembly and colloidal lithography, offer scalability and cost-effectiveness but typically face challenges in achieving high structural fidelity and uniformity over large areas. Conversely, top-down methods, including photolithography and electron-beam lithography, enable precise pattern definition but are often time-consuming, equipment-intensive, and associated with high operational costs, limiting their use to small-area or prototype devices. Recent efforts have explored hybrid strategies that combine bottom-up self-assembly with top-down patterning to leverage the advantages of both; yet, such approaches often introduce additional processing complexity and alignment challenges. Consequently, despite significant progress, a low-cost, high-throughput, and high-fidelity fabrication technique capable of faithfully replicating hierarchical compound-eye structures over large areas remains an unmet need.

Among these techniques, replication-based molding methods are particularly favored for preparing compound-eye arrays due to their ability to combine high replication fidelity with excellent scalability, making them well-suited for practical and industrial applications [19]. Among the various replication-based approaches, Yanagishita et al. demonstrated the use of anodic porous alumina molds to fabricate antireflection structures on pre-formed polymer lenses via nanoimprinting. While that work successfully transferred nanoporous features onto curved lens surfaces, the process required pre-existing lenses as the substrate and focused solely on the antireflection function [20]. In such processes, the quality of the final polymer replica is largely determined by the mold insert, which must faithfully replicate negative compound-eye features with high precision, durability, and resistance to repeated thermal and mechanical cycling [21,22]. However, fabricating such high-quality mold inserts using traditional bottom-up or top-down approaches remains challenging. Conventional methods are often cost-prohibitive because they rely on expensive equipment and involve complex, multi-step processing sequences. Alternatively, they may be excessively time-consuming due to low throughput and the need for iterative optimization of processing parameters [23,24,25,26]. These limitations significantly hinder the industrial viability of current fabrication strategies, particularly for large-area or high-volume production. Therefore, there is an urgent and growing need for an efficient, low-cost, and scalable method to fabricate mold inserts with high-precision compound-eye structures, thereby enabling the mass production of bioinspired optical surfaces for widespread technological applications in areas such as photovoltaics, displays, and optical sensing.

Herein, an injection compression molding (ICM) strategy is proposed to enable facile and high-fidelity fabrication of the aforementioned bioinspired compound-eye structure. Unlike conventional multi-step lithographic methods, the ICM process integrates mold filling and compression into a single cycle, allowing rapid replication of both micro- and nanoscale features. Using this strategy, polystyrene (PS) replicas featuring orderly and densely packed compound-eye arrays were fabricated within a cycle time of approximately 50 s. The simultaneous replication of both microlens arrays and nanopillars in a single ICM step. The resulting replicas exhibit both pronounced hydrophobicity and antireflectivity.

## 2. Materials and Methods

### 2.1. Materials

PS resin with the grade designation N1841H, a general-purpose thermoplastic known for its good melt flowability and high replication fidelity, was purchased from Hong Kong Petrochemical Co., Ltd., Hong Kong, China. An electro-polished aluminum plate with a thickness of 500 µm and a purity of 99.99% was purchased from Shangmu Tech., Shang Hai, China. All reagents used in the anode oxidation process, including oxalic acid, phosphoric acid, and other electrolytes, were purchased from Macklin Inc., Shang Hai, China. and used as received without further purification.

### 2.2. Characterization

The surface topographies of the PS microlens arrays, abbreviated as PS MLAs, the aluminum template, and the PS replicas were characterized using scanning electron microscopy (SEM). A Nova NanoSEM 430 instrument from FEI Co., Hillsboro, USA. The Netherlands, was employed for this purpose. Before imaging, all samples were sputter-coated with a thin gold layer to enhance conductivity and avoid charging effects. The reflectance spectra of the flat PS counterpart, the PS MLAs, and the PS replica surfaces were measured using a UV/VIS/NIR spectrometer, model UV3150 from Shimadzu Corp., Kyoto, Japan, which was equipped with an integrating sphere attachment to capture both specular and diffuse reflection components. The integrating sphere captures both specular and diffuse reflection components, providing the total reflectance. All reflectance measurements were conducted over the wavelength range from 400 nm to 1000 nm at normal incidence under ambient conditions. The wettability of the samples was evaluated using an automatic contact-angle tester equipped with a temperature-controlled stage, model OCA 40 from Data Physics Corp., Stuttgart, Germany. Deionized water droplets with a volume of 4 μL were gently deposited onto the sample surfaces at room temperature, and the static water CA was recorded after droplet stabilization. At least five different positions on each sample were measured to obtain an average value. Photothermal images captured during the durability tests were taken using an infrared thermal imager, model Fotric 226s from Fotric Inc., Shang Hai, China. This imager provides a thermal sensitivity of 0.05 °C and a frame rate of 25 Hz, enabling real-time monitoring of surface temperature changes under illumination or electrical heating. The wettability and wetting stability of the PS replica at elevated temperatures were measured at six discrete temperature points: 25 °C, 40 °C, 50 °C, 60 °C, 70 °C, and 80 °C, corresponding to temperature increments of 10 °C after the initial step. At each target temperature, the sample was allowed to equilibrate for five minutes to ensure a uniform surface temperature, which was verified by an integrated thermocouple in contact with the stage surface.

### 2.3. Fabrication of Compound-Eye Arrays

**Figure 1:**
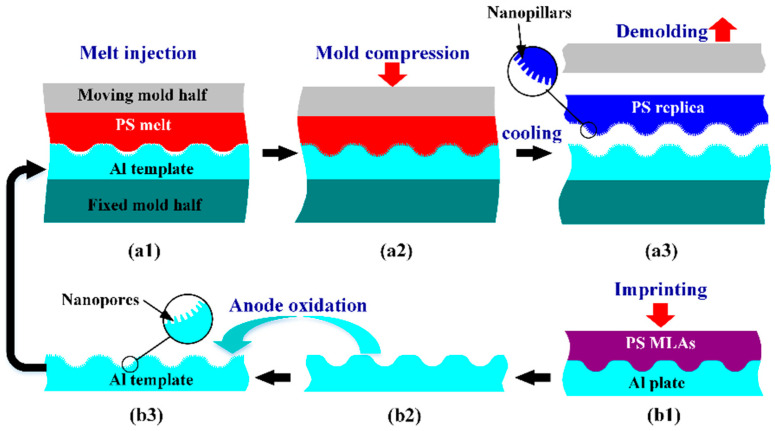
Fabrication process of PS replicas with compound-eye array. (**a1**–**a3**) ICM process for molding PS replica with compound-eye array; (**b1**,**b2**) imprinting MLAs onto an aluminum plate, and (**b3**) forming nanopores on the aluminum plate by using anode oxidation. Moreover, a novel strategy combining imprinting with anode oxidation, as illustrated in Figure 1(**b1**–**b3**), was proposed to prepare the aforementioned aluminum template with the negative compound-eye array. The PS microlens arrays, referred to as MLAs, were molded by using ICM technology with a flexible insert mounted on the mold cavity surface, and these MLAs were then used as a template for the imprinting process, as detailed in Supporting Note S1.

The PS replicas with the compound-eye array on their surfaces were molded via the ICM technology, as illustrated in Figure 1(a1–a3). PS was used as the molding material due to its sufficient melt flowability, low shrinkage, and high replication fidelity for micro/nanoscale features. The ICM setup consisted of an 80-ton injection molding machine (KM80SP180CX, KraussMaffei, Munich, Germany) and a mold equipped with a temperature control unit. An aluminum template featuring a negative compound-eye array was mounted on the cavity surface. The aluminum template was chosen for its high thermal conductivity, uniform surface energy, and excellent durability under cyclic compression. During the ICM process, the mold cavity was first partially filled with the PS melt under a partially closed condition, as shown in Figure 1(a1). This partial filling reduces flow resistance and minimizes shear-induced molecular orientation, which is beneficial for preserving the fidelity of fine structures. The mold was then compressed in the thickness direction to complete filling and to force the melt into the nanopores of the aluminum template, as illustrated in Figure 1(a2). The compression action not only ensures complete replication of both micro-lenses and nanopillars but also eliminates internal voids and weld lines. Subsequently, the melt was held under compression while being cooled, as depicted in Figure 1(a3), which suppresses post-shrinkage and maintains structural integrity. The key processing parameters were maintained constant: melt temperature at 240 °C, which is well above the glass transition temperature of PS to ensure low viscosity; injection rate at 154 cm^3^ s^−1^ and a mold compression speed of 35 mm/s, fast enough to fill the cavity before freezing but not so fast as to cause air entrapment; compression force at 280 kN, sufficient to push the melt into nano-scale cavities without damaging the aluminum template; and mold temperature at 120 °C, high enough to delay solidification allowing complete filling of nanostructures. For comparison, polystyrene counterparts without surface structures were prepared under the same ICM conditions. Notably, the total molding cycle time for each replica was approximately 50 s, which is substantially shorter than traditional hot embossing or lithographic methods, highlighting the potential for mass production. For comparison, polypropylene replicas with smooth surfaces were prepared under identical conditions but without the aluminum template, serving as a reference for evaluating the optical and wetting performance imparted by the compound-eye structures. The high reproducibility of the ICM process was confirmed by repeated molding of over 50 replicas without observable degradation of the aluminum template or loss of pattern fidelity.

Using a molding machine, the MLAs were imprinted onto an ultra-pure aluminum plate with a thickness of 0.5 mm to form a negative feature of the MLAs on the plate surface, as shown in Figure 1(b1,b2). The prepared aluminum plate was then pretreated following the procedure described in Supporting Note S2. Subsequently, anode oxidation was performed to form nanopores on the plate surface, as depicted in Figure 1(b3), where the pretreated aluminum plate served as the working electrode and a stainless-steel plate served as the counter electrode. Further details of both the pretreatment and anode oxidation processes are provided in Tables S1 and S2.

## 3. Results and Discussion

### 3.1. Topography and Antireflectivity

**Figure 2:**
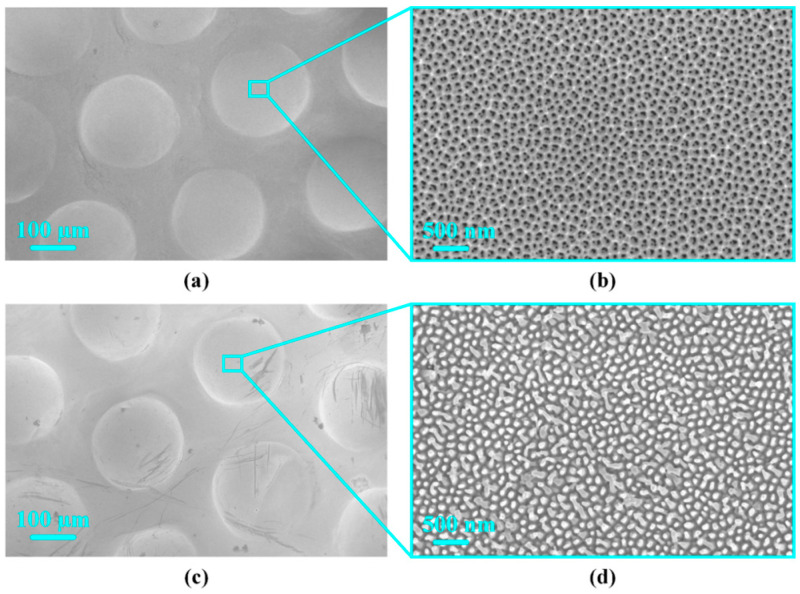
(**a**) Negative microlens and (**b**) nanopores on aluminum template surface, and (**c**) microlens and (**d**) nanopillars on PS replica surface.

The surface topographies of the PS MLAs, aluminum template, and PS replicas were characterized using SEM. As shown in Figure S1, the PS MLA surface exhibits orderly and periodically distributed microlenses with an average diameter of approximately 190 μm and an average pitch of approximately 320 μm. This regular arrangement is essential for achieving uniform optical and wetting properties across the entire surface. The microscale features on the PS MLAs are accurately transferred onto the aluminum plate via the imprinting process, as illustrated in Figure 2a. The imprinting step ensures faithful replication of the convex microlens pattern into a concave negative form on the aluminum surface. After the subsequent anode oxidation treatment, dense nanopores with an average diameter of approximately 80 nm and an average pitch of approximately 200 nm are formed on the aluminum template, as shown in Figure 2b. These nanopores are uniformly distributed over the concave microlens surfaces as well as the flat areas between them. Consequently, the prepared aluminum template possesses a negative compound-eye array feature that integrates both microscale concave lenses and nanoscale pores. The convex microlens with an average diameter of approximately 225 μm and an average pitch of approximately 320 μm is orderly distributed on the molded PS replica surface, as depicted in Figure 2c. The microlens diameter increases from approximately 190 μm in the original PS MLA to approximately 225 μm in the final PS replica. This enlargement is attributed to two consecutive effects during template fabrication: mechanical imprinting of the MLA into the aluminum plate causes plastic deformation of the soft metal, resulting in concave cavities that are slightly larger than the original convex features (Note S3). More interestingly, dense nanopillars with an average diameter of approximately 63 nm are formed not only on the convex microlens surfaces but also on the basement surface between the microlenses, as revealed in Figure 2d. These nanopillars arise from the faithful filling of the nanopores in the aluminum template by the PS melt during the injection compression molding process. The quantitative statistical data of the aluminum template, PS MLA, and PS replica are shown in Table S1. Thus, the microscale and nanoscale features of the aluminum template are accurately replicated onto the PS surface using ICM, resulting in the formation of a dual-level compound-eye array on the PS replica. This hierarchical structure closely mimics the natural compound eye and is responsible for the exceptional hydrophobicity and antireflectivity demonstrated by the replicas.

**Figure 3:**
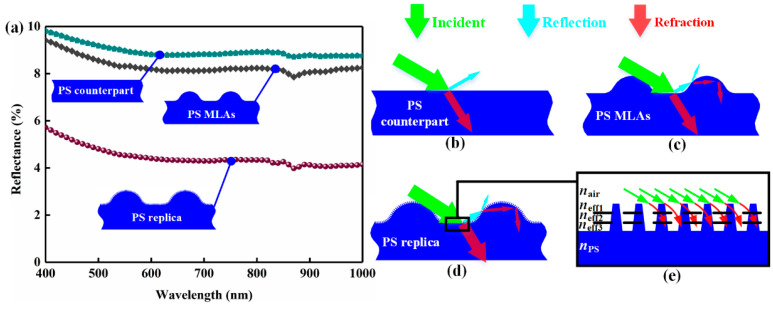
(**a**) Reflectance spectra of PS counterpart, PS MLA, and PS replica surfaces and mechanisms for reflectance on (**b**) PS counterpart, (**c**) PS MLA, and (**d**,**e**) PS replica surfaces.

**Figure 4:**
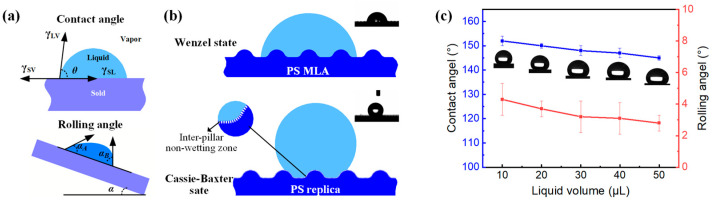
(**a**) Graphical definitions of contact and rolling angles. (**b**) Wetting states for 4 μL water droplets and corresponding mechanisms on PS MLAs and PS replica surfaces. (**c**) CA and RA of different water droplets on the PS replica.

The reflectance spectra of the PS counterpart, the PS microlens array referred to as PS MLA, and PS replica surfaces were measured using a UV/VIS/NIR spectrometer equipped with an integrating sphere attachment. In the wavelength range of 400–1000 nm, the average reflectance of the PS counterpart, PS MLA, and PS replica is approximately 9%, 8%, and 4%, respectively (Figure 3a). The flat PS counterpart exhibits the highest reflectance due to the absence of any surface texturing. As illustrated in Figure 3b, when light is incident on the flat PS counterpart surface, Fresnel reflection occurs because of the abrupt refractive index mismatch between PS, with a refractive index of approximately 1.59, and air, with a refractive index of 1. This sharp interface causes a significant portion of incident light to be reflected, limiting optical transmission. The antireflective behavior of the structured surfaces is attributed to their distinct topographies. For the PS MLA, the convex semi-spherical microlenses induce total internal reflection at curved interfaces, which redirects a portion of reflected light back into the substrate, leading to a modest reduction in reflectance to 8%, as depicted in Figure 4c. However, the reduction is limited because the microlenses alone do not provide a continuous refractive index gradient. In contrast, the PS replica features nanopillars on both the microlens surfaces and the basal plane. This hierarchical structure can be approximated as a homogeneous layer with an effective refractive index that gradually transitions from that of air, denoted as nair, to that of PS, denoted as nPS. The presence of nanopillars introduces a porosity gradient from the top to the bottom of the nanostructured layer (Figure 3d). The volume fraction of PS increases, gradually raising the effective index to that of bulk PS (Figure 3e). A quantitative optical model based on effective medium theory and the transfer matrix method is developed in Note S4. Such a gradient refractive index profile virtually eliminates the abrupt optical discontinuity at the PS/air interface, thereby suppressing Fresnel reflection across a broad spectral range and over a wide range of incidence angles [27]. This significant reduction in reflectance, from 9% of the flat counterpart to 4% of the biomimetic replica, corresponds to a relative decrease of over 55%, highlighting the superior antireflective performance enabled by the hierarchical compound-eye architecture.

### 3.2. Wettability and Durability

The wettability of MLAs and PS replicas was evaluated using an automatic contact-angle tester. Deionized water droplets with a volume of 4 μL were gently deposited onto the sample surfaces at ambient temperature, and the CA and RA were recorded after droplet stabilization (Figure 4a). As shown in Figure 4b, the PS MLA surface yields a CA of 86 ± 2°, whereas the PS replica surface exhibits a significantly higher CA of 151 ± 2°. The former value is characteristic of a hydrophilic surface, while the latter falls into the superhydrophobic regime. The hydrophilic nature of the PS MLA arises from its low aspect ratio semi-spherical microlenses. Because the microlenses are shallow and widely spaced, they cannot effectively trap air beneath the water droplet. Consequently, the droplet penetrates the inter-lens valleys, establishing a large solid–liquid contact area. This wetting behavior is well described by the Wenzel model, where surface roughness amplifies the intrinsic hydrophilicity of PS [28]. By contrast, the outstanding hydrophobicity of the PS replica is conferred by its compound eye hierarchical micro/nanostructures. Specifically, dense nanopillars are uniformly present on both the convex microlens surfaces and the flat basal plane. When a water droplet rests on such a dual-scale topography, the nanopillars create numerous air pockets that prevent the droplet from fully contacting the solid surface. The droplet thus rests primarily on the tips of the nanopillars, drastically reducing the solid–liquid contact area. This wetting regime is well described by the Cassie–Baxter model, where the apparent CA is governed by the area fraction of solid in contact with the liquid. The specific wetting mechanism is developed in Note S5. In the present case, the measured CA confirms that the surface is in a stable Cassie–Baxter state, which is highly desirable for self-cleaning, anti-icing, and drag reduction applications [29].

Further measurements were conducted to evaluate the wetting behavior of water droplets with varying volumes on the PS replica surface. Droplets with volumes ranging from 10 μL to 50 μL were gently deposited onto the surface using a precision syringe, and both the CA and the RA were recorded at room temperature using the automatic contact-angle tester. As shown in Figure 4b, as the droplet volume increases, the CA gradually decreases from approximately 151° to 146°, yet it consistently remains well within the hydrophobic regime. This slight decrease in CA with increasing droplet volume can be attributed to the gravitational effect: larger droplets exert higher gravitational pressure, causing partial penetration of the liquid into the micro/nanostructures and slightly reducing the apparent CA. Nevertheless, the hierarchical structure remains effective in trapping air pockets, preventing the transition to a fully wetted Wenzel state. Meanwhile, RA, which is defined as the minimum tilt angle at which a droplet begins to move on the surface, also exhibits a gradual decline with increasing droplet volume. Specifically, the RA drops from approximately 4° for a 10 μL droplet to about 2° for a 50 μL droplet. A lower RA indicates easier droplet mobility, which is beneficial for self-cleaning and anti-icing applications. The decrease in RA with volume is explained by the fact that larger droplets possess a greater gravitational driving force relative to the adhesion force at the solid–liquid interface. Overall, these results confirm that the PS replica surface maintains robust hydrophobicity and excellent droplet shedding capability across a wide range of droplet volumes, further validating its practical utility in outdoor and agricultural applications where droplet sizes vary considerably.

**Figure 5:**
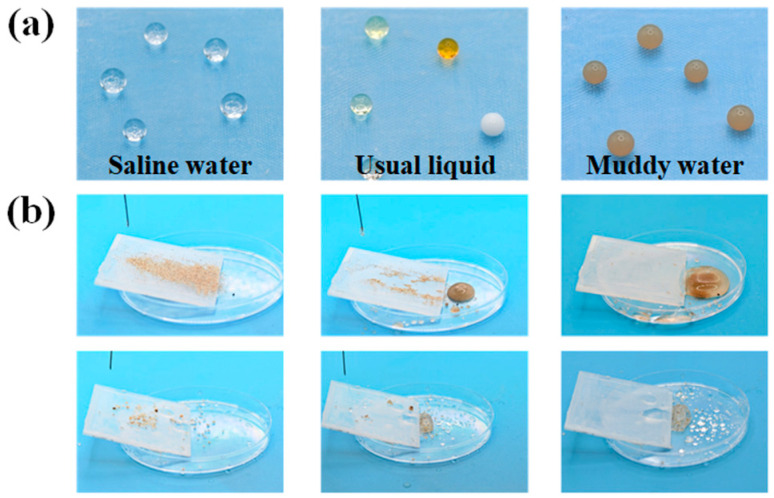
(**a**) Wetting state of different liquids and (**b**) self-cleaning property on the PS replica.

Subsequently, the practical performance of the PS replica surface was evaluated. As shown in Figure 5a, the surface remained hydrophobic when exposed to a range of challenging liquids, including a 3.5% saline solution simulating seawater, common household liquids such as tea, juice, and milk, as well as muddy water containing fine soil particles. In all cases, water droplets maintained a hydrophobic state, indicating that the hierarchical micro/nanostructure effectively prevented liquid spreading and penetration. Furthermore, the self-cleaning capability of the PS replica was investigated by depositing sand particles of different sizes (Figure 5b). When a falling water droplet impacted the contaminated surface, it was observed that the droplet readily rolled off and simultaneously carried away the sand particles. This behavior is attributed to the low adhesion force between the solid surface and both the liquid and the contaminant. Because the surface is in a stable Cassie–Baxter state with trapped air pockets, the contact area and the resulting adhesion are minimized. Consequently, the kinetic energy of the rolling droplet is sufficient to detach and entrain the sand grains, leaving a clean surface behind. Quantitative self-cleaning removal efficiency and durability are shown in Table S5 and Note S6. The average removal efficiency over 10 cleaning cycles was approximately 92%, with no significant decrease observed with increasing cycle number. This water-driven removal of particulate contaminants clearly demonstrates the outstanding self-cleaning property of the PS replica. Taken together, these results confirm that the PS replica not only resists wetting by a wide variety of liquids but also effectively sheds solid contaminants with the help of moving water droplets [30]. Such dual functionality makes the biomimetic surface highly attractive for practical applications that require both liquid repellency and easy cleanability, including outdoor optical devices, agricultural films, and building facades.

**Figure 6:**
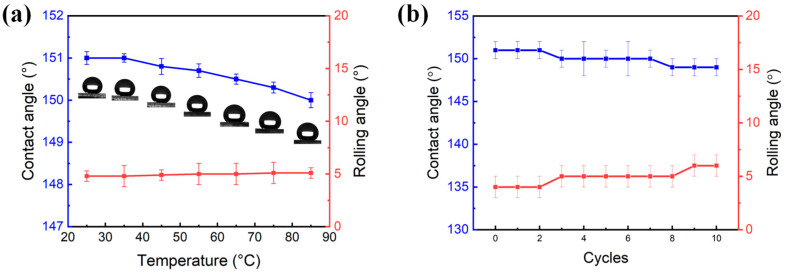
(**a**) Wetting stability of the PS replica surface at different temperatures and (**b**) cyclic heating test.

Furthermore, to verify the wetting stability of the PS replica surface at elevated temperatures, the CA and RA were systematically measured from 25 °C to 80 °C at increments of 10 °C using a CA meter equipped with a temperature-controlled stage. The results are shown in Figure 6a. At each temperature setting, the sample was held for 5 min to ensure thermal equilibrium before measurement, and at least five different positions on the surface were tested to obtain reliable average values. It can be observed that the CA of the sample surface decreases slightly with increasing temperature. This gentle decline is attributed to the reduced stability of air pockets trapped within the hierarchical micro/nanostructures at higher temperatures. As the temperature rises, the increased molecular motion and reduced surface tension of water promote gradual liquid penetration into the inter-nanopillar spaces, thereby partially disrupting the Cassie–Baxter state. Meanwhile, the RA shows a slight increase from roughly 4° at 25 °C to about 5° at 80 °C. This increase is due to the enlarged solid–liquid contact area resulting from the partial wetting transition, which enhances the adhesion force between the droplet and the surface. Despite these minor variations, the CA remains consistently above 150°, and the RA stays below 10° across the entire temperature range from 25 °C to 80 °C. These results unequivocally demonstrate the excellent high-temperature wetting stability of the PS replica surface. In contrast, conventional flat polymer surfaces typically lose their hydrophobic properties at much lower temperatures due to the absence of protective air pockets. The sustained superhydrophobicity of the biomimetic surface under elevated temperatures is therefore a key advantage for practical applications in hot climates or for devices that generate heat during operation, such as outdoor solar panels or optical sensors exposed to direct sunlight. To evaluate the stability of the superhydrophobic surface under repeated thermal cycling, the PS replica sample was subjected to 10 thermal cycles between 25 °C and 80 °C. Each cycle consisted of heating from 25 °C to 80 °C at a rate of 5 °C/min, holding at 80 °C for 30 min, cooling from 80 °C to 25 °C at a rate of 5 °C/min, and holding at 25 °C for 30 min. The water contact angle and rolling angle were measured at 25 °C after the completion of each cycle, and the results are shown in Figure 6b. After 10 thermal cycles, the CA remained at 149° ± 2°, and the RA remained at 6° ± 1°, indicating that the superhydrophobic properties are essentially retained. The slight decrease from 151° to 149° and the slight increase in RA from 4° to 6° are within experimental error and are attributed to minor thermally induced surface reorganization at the molecular level, rather than to structural collapse. These results confirm that the PS replica surface maintains superhydrophobic stability under repeated heating across the temperature range relevant to the study.

**Figure 7:**
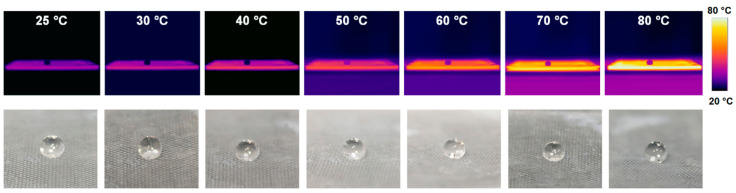
Infrared thermal images and the corresponding wetting states of water droplets on the PS replica surface.

The infrared thermal images and the corresponding wetting states of water droplets on the PS replica surface during the heating process are presented in Figure 7. The infrared thermal images record the temperature distribution of the droplet and the surrounding surface in real time. Within the temperature range from 25 °C to 80 °C, it is clearly observed that water droplets on the PS replica surface maintain a spherical or near-spherical shape, indicating a stable hydrophobic state. This behavior is also consistently reflected in the infrared thermal images. The droplets appear as distinct, well-defined cold spots against the warmer background of the heated surface, with sharp droplet boundaries that suggest minimal contact area and low thermal conduction between the droplet and the solid surface. As the temperature increases from 25 °C to 80 °C, no wetting transition occurs, and the droplets remain in the Cassie–Baxter state, as evidenced by the absence of spreading or flattening in both the optical and thermal images. The fact that the infrared thermal images corroborate the optical observations strongly confirms that the surface retains its superhydrophobic character even at elevated temperatures.

**Figure 8:**
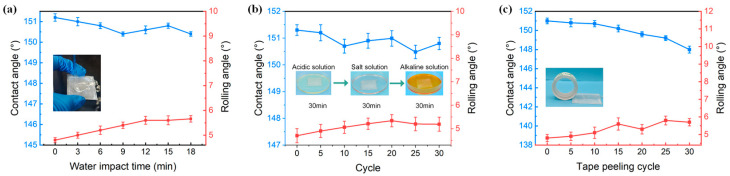
CA and RA in (**a**) water impacting cycles, (**b**) chemical resistance testing cycles, and (**c**) tape peeling cycles.

The durability of the PS replica surface was systematically evaluated through tape peeling, water impact, chemical resistance, and outdoor exposure tests. In the tape peeling test, the surface maintained a contact angle above 148° and a rolling angle below 6° after 30 cycles of 180° peeling with 3M Scotch tape, demonstrating excellent mechanical robustness attributable to the monolithic injection-molded structure (Figure 8a). The water impact test, performed with a water jet at a flow rate of approximately 1 mL/s from a height of 45 cm for 18 min, showed that the surface retained a contact angle above 149° and a rolling angle below 6°, confirming the high critical pressure of the Cassie–Baxter state (Figure 8b). Chemical resistance was evaluated by sequential immersion in acidic (pH 3.0), alkaline (pH 11.0), and saline (3.5 wt% NaCl) solutions, with the surface maintaining a contact angle above 150° and a rolling angle below 6° after 30 cycles (Figure 8c). Furthermore, a 10-day outdoor exposure test under real-world conditions, including sunlight, rain, and temperature fluctuations, confirmed that the superhydrophobic properties were well preserved, with only a slight decrease in contact angle and a minor increase in rolling angle within acceptable ranges (Note S7, Tables S6 and S7). These comprehensive durability evaluations substantially strengthen the practical relevance of the PS replica surface for self-cleaning and optical applications.

## 4. Conclusions

By applying imprinting and anode oxidation, the negative MLA feature and nanopores with an average diameter of approximately 80 nm are successfully formed on the aluminum template. The micro- and nano-scale features on the aluminum template are successfully replicated onto the PS surface by applying ICM technology. The dual-level compound-eye array consisting of convex semi-spherical microlenses with an average diameter of about 225 μm and dense nanopillars with an average diameter of about 63 nm is formed on the PS replicas. The nanopillars on the microlens and basement surfaces can effectively hinder the droplet from wetting the internanopillar areas, resulting in the hydrophobic Cassie–Baxter state with a CA of 151° ± 2° for the 4 μL water droplet. Furthermore, the PS replica surface maintains stable superhydrophobicity even at elevated temperatures up to 80 °C, with the CA remaining above 150° and the RA below 10°, demonstrating excellent high-temperature wetting stability. In addition, the nanopillars create a gradient refractive index that gradually transitions from that of air to that of bulk PS, which effectively suppresses Fresnel reflection across a broad wavelength range. Consequently, the PS replicas achieve an average reflectance as low as about 4% over the 400–1000 nm spectral range. Thus, the PS replicas exhibit both robust hydrophobicity and excellent antireflectivity, attributes that are directly enabled by their biomimetic compound-eye architecture. By using the aluminum template with the negative compound-eye array to replace traditional mold inserts fabricated by conventional bottom-up or top-down methods, this approach offers an efficient route for producing bioinspired polymer compound-eye arrays via injection molding.

## Data Availability

The original contributions presented in this study are included in the article. Further inquiries can be directed to the corresponding authors.

## Supplementary Materials

The following supporting information can be downloaded at https://www.mdpi.com/article/10.3390/biomimetics11070507/s1. Note S1: Micro-lens arrays molded by injection compression molding; Note S2: Detailed treatment and anode oxidation of aluminum template. Note S3: Quantitative statistics of the template and replica. Note S4: Quantitative optical model based on effective medium theory. Note S5: Specific wetting mechanism. Note S6: Quantitative self-cleaning removal efficiency. Note S7: The durability of the PS replica surface.

## Abbreviations

The following abbreviations are used in this manuscript:

CA	Contact angle
RA	Rolling angle
MLAs	Microlens arrays
ICM	Injection compression molding
