# Supplementary material for: Rapid Fabrication of Bioinspired Compound-Eye Array with Hydrophobicity and Antireflectivity
Source: Biomimetics (Basel). 2026 Jul 19;11(7):507. doi: 10.3390/biomimetics11070507 (PMC13407124; doi:10.3390/biomimetics11070507)
Supplement: Supplementary file 1 [file biomimetics-11-00507-s001.zip › biomimetics-4369535-supplementary.pdf]

Supporting Information for

Rapid fabrication of bioinspired compound-eye array with hydrophobicity and antireflectivity

Zirui Yao[1], Lelai Yuan[2], Jiabao Lu[2], Gang Huang[1], Zihao Li[2], Yu Li[1], Heng Xie[2]* and Guizhen Zhang[1]*

[1] National Engineering Research Center of Novel Equipment for Polymer Processing, School of Automation Science and Engineering, South China University of Technology, Guangzhou 510640, P. R. China.

[2] Hubei Key Laboratory of Plasma Chemistry and Advanced Materials; School of Materials Science and Engineering, Wuhan Institute of Technology, Wuhan 430205, P. R. China.

*Corresponding authors:

Heng Xie E-mail: hengxie@hust.edu.cn

Guizhen Zhang E-mail: guizhenzhang@scut.edu.cn

Note S1: Microlens arrays molded by injection compression molding

As shown in Figure S1, the microlenses with a diameter of about 190 μm and a pitch of about 320 μm are distributed orderly and periodically throughout the molded MLAs surface. The micro-lens feature was imprinted into the aluminum sheet in the master mold preparation process.

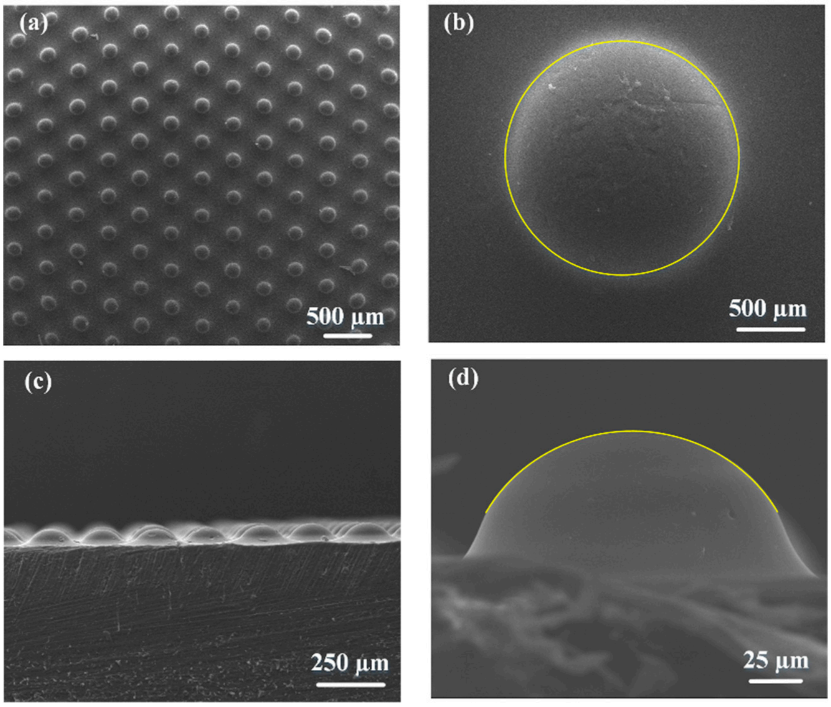

Figure S1 (a, b) SEM images of MLA and (c, d) side view of single micro-lens.

Note S2: Detailed treatment and anode oxidation of aluminum template

The prepared Al plate with microlens feature was pretreated according to the procedure outlined in Table S1. Then, anode oxidation was performed with the pretreated aluminum sheet as working electrode and stainless steel as counter electrode. The Detailed steps of the anode oxidation process are shown in Table S2. The electrode spacing is 8.5cm. A power supply (IT7000 DC Power Supply, ITECH Electronic Co. Ltd, USA) was used to provide a constant current density of 15 mA/cm^2 for the first anode oxidation process. Then, the same power supply was used to provide a constant voltage of 55 V for the second anode oxidation process.

Table S1 Detailed treatment processes of aluminum plate with MLAs features.

Processes	Composition	Concentration	Condition
Ultrasonic degreasing	Ethyl alcohol absolute (C_2H_6O)	99.9%	RT, 10 min
Alkaline etching	Sodium hydroxide (NaOH)	1 mol/L	RT， 3 min
Ultrasonic cleaning	Deionized water	/	RT, 20 min
	Phosphoric acid (H_3PO_4)	500 ml/L	
Chemical polishing	Sulfuric acid (H_2SO_4, 98%)	400 ml/L	80°C， 10 min
	Nitric acid(HNO_3, 68%)）	100 ml/L	
Ultrasonic cleaning	Deionized water	/	
Vacuum drying	/	/	80°C, 30 min

Table S2 Detailed steps of anode oxidation process.

Steps	Composition	Concentration	Condition
First anode oxidation	Citric Acid($C_6H_8O_7$)	30 g/L	6 °C, 60 min
	Phosphorous acid (H_3PO_3)	10 g/L	
Ultrasonic cleaning	Deionized water	\	RT, 10 min
Acid etching	Chromium trioxide (CrO_3)	20 g/L	RT, 180 min
	Phosphoric acid (H_3PO_4, 85%)	50 ml/L	
Ultrasonic cleaning	Deionized water	\	
Second anode oxidation	Oxalic acid ($H_2C_2O_4$)	4 mol/L	15 °C, 20 min
Ultrasonic cleaning	Deionized water		RT, 10 min
Vacuum drying	\	\	80 °C, 30 min

Note S3: Quantitative statistics of the template and replica

The microlens diameter increases from approximately 190 μm in the original PS MLA to approximately 225 μm in the final PS replica. This enlargement is attributed to two consecutive effects during template fabrication: (i) mechanical imprinting of the MLA into the aluminum plate causes plastic deformation of the soft metal, resulting in concave cavities that are slightly larger than the original convex features; and (ii) subsequent anodic oxidation consumes the aluminum surface and further widens the pore openings. Importantly, the injection compression molding step itself, from the aluminum template to the PS replica, achieves a replication ratio of approximately 1.02, confirming that the ICM process provides high-fidelity transfer of the template geometry.

Moreover, quantitative statistical data of the PS MLA, aluminum template, and PS replica have been compiled from re-analysis of the existing SEM micrographs (Table S3).

Table S3 Quantitative statistics of the template and replica.

Feature	Parameter	PS MLA	Aluminum template	PS replica
Microlens	Diameter (μm)	190 ± 10	220 ± 10	225 ± 12
	Pitch (μm)	320 ± 15	320 ± 12	320 ± 18
Nanopillar	Diameter (nm)	/	80 ± 8	63 ± 7
	Pitch (nm)	/	200 ± 15	200 ± 18
	Height (nm)	/	~250	~250

Note S4 Quantitative optical model based on effective medium theory

To compensate for the lack of angle-dependent measurements, a quantitative optical model based on effective medium theory (EMT) and the transfer matrix method (TMM) has been developed.

1. Volume fraction calculation

The nanopillar array is discretized into N discrete layers along the height direction (N=20). Each layer i can be treated as a composite medium consisting of PS and air. For each layer, the volume fraction of PS, $f_{PS}(z_i)$, is determined by the geometry of the nanopillars.

For cylindrical nanopillars, the PS volume fraction at height z is given by:

$$f_{PS}(z) = \frac{\pi[d(z)/2]^2}{P^2} \tag{S1}$$

Since the nanopillars exhibit an approximately constant diameter from bottom to top (cylindrical shape), and the slight rounding at the tips is neglected, the following applies:

$$f_{PS} = \frac{\pi(63/2)^2}{200^2} \approx 0.078 \tag{S2}$$

Along the height direction, from the nanopillar tips (z=h) to the substrate (z= 0), f_{PS} transitions from near zero (at the tips, where only the nanopillar apex exists) to 0.078 (within the pillar body), and then to 1.0 (at the substrate, corresponding to the continuous PS layer). To construct the graded-index profile, a linear interpolation model is employed:

$$f_{PS}(z) = \begin{cases} 0.078 \times \left(\frac{z}{h}\right), & 0 \leq z \leq h \ (pillar\ region) \\ 1.0, \quad z < 0 & (substrate\ continuous\ layer) \end{cases} \tag{S3}$$

A transition layer with a very low filling fraction is additionally introduced at the top to simulate the graded interface between the nanopillar tips and air.

2. Effective refractive index calculation

For each layer, the Bruggeman effective medium approximation is applied to solve for n_{eff}:

$$f_{PS}\frac{n_{PS}^2-n_{eff}^2}{n_{PS}^2+2n_{eff}^2}+(1-f_{PS})\frac{n_{air}^2-n_{eff}^2}{n_{air}^2+2n_{eff}^2}=0 \quad \text{(S4)}$$

Let $x=n_{eff}^2, a=n_{PS}^2=2.5281, and\ b=n_{air}^2=1.00.$ The equation becomes:

$$f_{PS}\frac{a-x}{a+2x}+(1-f_{PS})\frac{b-x}{b+2x}=0 \quad \text{(S5)}$$

Multiplying through by the common denominator, collecting like terms, and substituting a=2.5281 and b=1:

$$2.5281+(-0.5281+4.5843f_{PS})x-2x^2=0 \quad \text{(S6)}$$

Solving this quadratic equation and taking the positive root:

$$x=\frac{(-0.5281+4.5843f_{PS})+\sqrt{(-0.5281+4.5843f_{PS})^2+20.2248}}{4} \quad \text{(S7)}$$

Thus, $n_{eff}=\sqrt{x}$. The representative values of volume fractions and effective refractive indices for each layer are listed in Table S4.

Table S4 Values of volume fractions and effective refractive indices for each layer.

Layer *i*	*z*/*h*	f_{PS}	n_{eff}
1	1.00	0.005	1.012
2	0.90	0.015	1.036
3	0.80	0.025	1.060
4	0.70	0.035	1.085
5	0.60	0.045	1.109
6	0.50	0.055	1.134
7	0.40	0.065	1.158
8	0.30	0.075	1.183
9	0.20	0.078	1.187
10	0.10	0.078	1.187
11	0.00	0.078	1.187
Substrate	<0	1.000	1.590

From the top to the bottom, the effective refractive index transitions continuously from approximately 1.01 to 1.59, achieving impedance matching.

3. Transfer matrix method reflectance calculation

For the i-th layer, the phase thickness is:

$$\delta_i=\frac{2\pi}{\lambda}n_{eff},it_i \quad \text{(S8)}$$

where t_i=h/N, and λ is the wavelength of incident light in vacuum. The characteristic matrix of the i-th layer is:

$$M_i = \begin{pmatrix} cos\delta i & \frac{isin\delta_i}{\eta_i} \\ i\eta_i sin\delta_i & cos\delta_i \end{pmatrix} \quad \text{(S9)}$$

where $\eta_i = n_{eff,}\ i$ (optical admittance at normal incidence).

The total transfer matrix for the entire multilayer system is the product of the matrices of all layers:

$$M = M_1 M_2 M_3 \cdots M_N \quad \text{(S10)}$$

For $N = 20$ layers, this expands to:

$$M = \prod_{i=1}^{N} \begin{pmatrix} \cos\delta_i & \frac{i\sin\delta_i}{\eta_i} \\ i\eta_i \sin\delta_i & \cos\delta_i \end{pmatrix} \quad \text{(S11)}$$

The total matrix is expressed as:

$$M = \begin{pmatrix} M_{11} & M_{12} \\ M_{21} & M_{22} \end{pmatrix} \quad \text{(S12)}$$

The optical admittance of the incident medium (air) is $\eta_0 = n_{air} = 1.00$, and that of the substrate (PS) is $\eta_s = n_{PS} = 1.59$.

The reflection coefficient (amplitude) is:

$$r = \frac{(M_{11}+M_{12}\eta_s)\eta_0-(M_{21}+M_{22}\eta_s)}{(M_{11}+M_{12}\eta_s)\eta_0+(M_{21}+M_{22}\eta_s)} \quad \text{(S13)}$$

The reflectance is:

$$R = | r |^2 = | \frac{(M_{11}+M_{12}\eta_s)\eta_0-(M_{21}+M_{22}\eta_s)}{(M_{11}+M_{12}\eta_s)\eta_0+(M_{21}+M_{22}\eta_s)} |^2 \quad \text{(S14)}$$

The above calculation is repeated at 5 nm intervals over the wavelength range of 400–1000 nm, yielding the reflectance spectrum $R(\lambda)$. The average reflectance is:

$$\bar{R} = \frac{1}{600}\int_{400}^{1000} R(\lambda)d\lambda \approx \frac{1}{N_\lambda}\sum_{j=1}^{N_\lambda} R(\lambda_j) \quad \text{(S15)}$$

The calculated results show an average reflectance of approximately 3.8%–4.2% across the 400–1000 nm range. Therefore, the TMM calculation results are in agreement with the experimental data, validating the graded-index antireflection mechanism. The nanopillar array is the key factor responsible for the significant reflectance reduction, as its graded-index effect effectively suppresses Fresnel reflection.

Note S5: Specific wetting mechanism

The intrinsic (Young) contact angle of a flat PS surface, θ_Y, was measured using a smooth PS film prepared under identical molding conditions but without the aluminum template. The measured value is 86°±2°. This value serves as the reference for both the Wenzel and Cassie-Baxter model

calculations.

For the PS MLA, the surface consists of hemispherical microlenses with diameter D=190 μm and pitch P=320 μm. The roughness factor r is defined as the ratio of the actual surface area to the projected area:

$$r = \frac{A_{actual}}{A_{projected}} \quad \text{(S16)}$$

For a hemispherical cap, the surface area of a single microlens is:

$$A_{\text{cap}} = 2\pi(\frac{\text{D}}{2})^2 \quad \text{(S17)}$$

The projected area of a single unit cell is:

$$\text{A}_{\text{projected}}=P^2 \quad \text{(S18)}$$

Assuming the microlenses are arranged in a square lattice, the roughness factor is:

$$\text{r} = 1 + \frac{\text{A}_{\text{cap}}}{\text{A}_{\text{projected}}} = 1 + \frac{56706}{102400} = 1 + 0.554 = 1.554 \quad \text{(S19)}$$

The Wenzel equation relates the apparent contact angle θ_{W} to the intrinsic contact angle θ_{Y} through the roughness factor r:

$$cos\,\theta_{\text{W}} = \text{r}cos\,\theta_Y \quad \text{(S20)}$$

Substituting the values:

$$cos\,\theta_{\text{W}} = 1.554 \times cos\,86° = 1.554 \times 0.06976 = 0.1084 \quad \text{(S21)}$$

$$\theta_{\text{W}} = arccos\,(0.1084) = 83.8° \quad \text{(S22)}$$

The calculated apparent contact angle (83.8°) is in excellent agreement with the experimentally measured value of 86° $\pm$ 2° for the PS MLA surface. This confirms that the water droplet on the PS MLA adopts the Wenzel state, where the liquid fully penetrates the microlens valleys.

For the PS Replica with compound eye array, the nanopillar diameter and pitch are 63 nm and 200 nm, respectively. For cylindrical nanopillars arranged in a square lattice, the solid fraction f (the fraction of the solid surface in contact with the liquid) is:

$$f = \frac{\pi(\text{d}/2)^2}{\text{P}^2} = \frac{\pi\times(63/2)^2}{(200)^2} = \frac{\pi\times31.5^2}{40000} = \frac{3117}{40000} = 0.0779 \approx 0.078 \quad \text{(S23)}$$

The Cassie-Baxter equation for a composite solid–air interface is:

$$cos\ \theta_{\text{CB}} = \text{f}(cos\ \theta_{\text{Y}} + 1) - 1 \quad \text{(S24)}$$

Substituting the values:

$$\theta_{\text{CB}} = arccos\,(-0.9166) = 153.5° \quad \text{(S25)}$$

The calculated apparent contact angle matches the experimentally measured value of 151° $\pm$ 2° within experimental error. This confirms that the water droplet on the PS replica surface adopts the Cassie-Baxter state, with the droplet resting predominantly on the tips of the nanopillars and trapped air pockets beneath it. The low hysteresis indicates weak adhesion between the water

droplet and the PS replica surface, which is consistent with the Cassie-Baxter state and explains the low rolling angle reported in the manuscript. The low hysteresis also suggests that the droplet can easily roll off the surface, which is beneficial for self-cleaning applications.

Note S6: Quantitative self-cleaning removal efficiency

A model contaminant was uniformly dispersed onto the PS replica surface at an areal density of approximately 0.5 g/m². The contaminated surface was then inclined at 10°, and a 50 μL water droplet was released from a height of 5 mm above the top edge of the inclined surface, allowing it to roll down across the contaminated area. This procedure was repeated for 10 cleaning cycles on the same surface area. The number of particles remaining on the surface was quantified by optical microscopy image analysis before and after each cleaning cycle. The contamination removal efficiency was calculated as:

$$\text{Removal Efficiency (\%)} = \left(1 - \frac{N_{after}}{N_{before}}\right) \times 100\% \tag{S26}$$

where N_{before} and N_{after} are the number of particles counted in the same observation area before and after cleaning. The results are shown in Table R5. The average removal efficiency over 10 cleaning cycles was approximately 92%, with no significant decrease observed with increasing cycle number. This confirms that the PS replica surface exhibits highly efficient and reproducible self-cleaning performance.

Table S5 Contamination removal efficiency in cleaning cycle.

Cleaning cycle	Particles counted (before)	Particles counted (after)	Removal efficiency (%)
1	285	18	93.7
2	292	22	92.5
3	278	20	92.8
4	301	25	91.7
5	288	23	92.0
10	295	28	90.5

Note S7: Durability of the PS replica surface

To evaluate the stability of the PS replica surface under real outdoor conditions, samples were placed on a flat rooftop exposure site (Wuhan, China, 30.6°N, 114.3°E) from June 15 to June 25, 2026. The samples were mounted on a platform at a 45° inclination facing south to maximize solar exposure while allowing rainwater drainage. The daily weather conditions during the test period were recorded, including temperature, relative humidity, and UV index. The CA and RA were measured daily at the same time for 10 consecutive days. The 10-day outdoor exposure test, conducted under real-world conditions including direct sunlight, rain, temperature fluctuations, and UV radiation, confirms that the PS replica surface maintains its superhydrophobic properties in a practical outdoor environment (Table S6). The slight decrease in CA and increase in RA 4°are within acceptable ranges for most self-cleaning applications (Table S7).

Table S6 Daily weather conditions during the test period.

Day	Date	Max Temp (°C)	Min Temp (°C)	RH (%)	UV Index
0	Jun 15	32	24	65	9
1	Jun 16	34	25	60	10
2	Jun 17	33	26	62	8
3	Jun 18	30	23	70	5
4	Jun 19	28	22	85	2
5	Jun 20	27	22	88	2
6	Jun 21	29	23	72	6
7	Jun 22	35	26	58	11
8	Jun 23	36	27	55	11
9	Jun 24	34	26	60	9
10	Jun 25	35	27	58	10

Table S7 CA and RA in the outdoor exposure testing process.

Exposure Day	Contact Angle (°)	Rolling Angle (°)
0	151 ± 2	4 ± 1
1	151 ± 2	4 ± 1
2	151 ± 2	4 ± 1
3	150 ± 2	4 ± 1
4	150 ± 2	5 ± 1

Exposure Day	Contact Angle (°)	Rolling Angle (°)
5	150 ± 2	5 ± 1
6	150 ± 2	5 ± 1
7	149 ± 2	5 ± 1
8	149 ± 2	5 ± 1
9	148 ± 2	6 ± 1
10	148 ± 2	6 ± 1
